# Association Between Dietary Supplement Use and Academic Achievement Among University Students in the Kingdom of Saudi Arabia: A Cross-Sectional Study

**DOI:** 10.7759/cureus.77378

**Published:** 2025-01-13

**Authors:** Suzan A Morsy, Ahmad S Alkamal, Mahdi T Al-Nahdi, Abdualaziz M Abed, Anas Alfarra, Mohammad Bantan, Abdullah Almotowa

**Affiliations:** 1 Clinical Pharmacology, Faculty of Medicine, Alexandria University, Alexandria, EGY; 2 Clinical Pharmacology, Fakeeh College for Medical Sciences, Jeddah, SAU; 3 Medicine, Fakeeh College for Medical Sciences, Jeddah, SAU

**Keywords:** academic achievement, adverse effects, caffeine, creatine, curcumin, dietary supplements, ginkgo biloba, omega-3 fatty acids, saudi arabia, students

## Abstract

Background

Dietary supplements are commonly utilized worldwide, including in the Kingdom of Saudi Arabia (KSA), primarily to improve memory, focus, wakefulness, learning, and academic achievement. Nevertheless, there is still uncertainty regarding their efficacy and safety, particularly concerning academic performance. This study examines the prevalence of dietary supplement use among students in KSA and how this affects their academic performance.

Methodology

The current study was a cross-sectional study conducted in KSA between 2022 and 2024 on a sample size of 513 students aged ≥18 years, as calculated by Epi Info software (Centers for Disease Control and Prevention, Atlanta, United States). A self-administered online questionnaire was designed, tested for validity and reliability, and then propagated via social media. It included four parts assessing the socio-demographic, past medical and medication history, academic information, and dietary supplement use.

Results

The results showed that 360 (70.2%) participants used dietary supplements, while most of them, 141 (39.2%), used a combination of supplements. Users who reported taking supplements every day were 266 (73.9% of supplement users), and the supplement-related adverse effects occurred in about 147 (41% of supplement users). The most commonly reported side effects were headaches, sleeplessness, and irritability. No discernible influence of any of the reported dietary supplements on academic achievement was identified, despite the high prevalence of supplement usage and the related negative effects. A high percentage of the participants had incorrect knowledge about dietary supplements - 262 (51.1%) had a false belief that supplements can improve their achievement and 238 (46.4%) considered them completely safe with no risks of adverse effects.

Conclusions

Nutritional supplement intake was highly prevalent among KSA students; however, no positive association was identified between academic achievement and any of the used supplements (caffeine, creatine, curcumin, omega-3 fatty acids, *Ginkgo biloba*, or multivitamins). False knowledge was common among the studied group about the effects of supplements on academic achievement and their safety.

## Introduction

The term "dietary supplement" refers to any food component, nutrient, or non-food chemical consumed with a regular diet to achieve a particular health and/or performance benefit [1]. They include plant extracts, vitamins, minerals, amino acids, and enzymes. The usage of dietary supplements has gained significant global popularity, particularly among students [2]. In 2019, the sales of these supplements in the Kingdom of Saudi Arabia (KSA) were approximately 875 million Saudi Riyals [3,4]. These supplements are frequently advertised as being able to improve concentration, cognitive function, and general wellness. The usage of dietary supplements has garnered special attention because academic performance is a major factor in determining a student's future chances [5].

Nonetheless, although certain research indicates possible advantages, other studies draw attention to the dangers of the irrational use of supplements and the absence of proof for numerous promoted claims [6,7]. Concerns have been raised regarding the potential impact of dietary supplements on academic performance [8]. Among the commonly used supplements is caffeine [9], which is advertised as a method to improve learning based on its known action to prolong the duration of wakefulness [10-12]. Other supplements marketed with similar claims of being brain supplements that improve memory and foster learning are creatine [13,14], curcumin [15], *Ginkgo biloba* [16], and omega-3 fatty acid [17,18]. Despite the common use of these supplements, their exact safety and efficacy are not completely elucidated, especially regarding their positive impact on the academic achievability of students.

The aim of this study was to assess the effect of dietary supplement intake on academic achievement among students in KSA. Additionally, it evaluated students' perception of the impact of the supplements on their academic achievement, safety, and effectiveness.

## Materials and methods

Sampling method, sample size calculation, and study design 

A cross-sectional study involving 513 students in KSA was carried out after determining the sample size using the Epi Info online calculator (Centers for Disease Control and Prevention, Atlanta, United States) at a confidence interval of 95% and 5% alpha error. The calculated sample size was 350 responses; however, it was expanded to include 513 participants to account for omissions and to lower the possibility of type II errors. The enrollment of the participants was done through a convenient sampling technique by sending links of the online questionnaire designed using Google Forms (Google Inc., Mountain View, United States).

Criteria for inclusion and exclusion

Criteria for inclusion: University students aged ≥18 years old, living in KSA, who had internet access to complete the questionnaire and could give consent were included in the study.

Criteria for exclusion: Individuals living outside KSA, having a chronic illness, or receiving chronic drug therapies were excluded from the study.

Data collection

This survey-based study used a self-administered questionnaire created via Google Forms and disseminated over social media platforms, including Facebook, Instagram, Snapchat, WhatsApp, Twitter, and others, starting from September 2022 to August 2024. The questionnaire began with a brief introductory part that was added to explain the aim of the research, clarify volunteer participation, and emphasize data anonymity and confidentiality and that it would not be used for any other reason than this study. All participants could reject participation or withdraw from completing the questionnaire at any point without declaring any reason or having a negative effect. The contact information of the investigators was added to the questionnaire introduction to be available for participants if they needed any clarification.

The 15 questions in the survey were broken down into four sections (see Table 7 in Appendices):

(i) Inquiries on the participants' age, sex, nationality, and place of residence comprised the first section. Responders who were living outside KSA were excluded from the results.

(ii) The second section asked about past medical and medication history and if the participant had any chronic illness or was on any chronic drug therapy. Those who had chronic illnesses or were using chronic medications were excluded from the study.

(iii) In the third section, questions about the participant’s academic information - type of study, academic year, and the average academic achievement of the last three years - were asked.

(iv) The fourth section inquired about the intake of any dietary supplement - if the participant was receiving any dietary supplement, the type of supplement received, duration, and frequency of intake, the occurrence of supplement-related adverse events, and the awareness about the impact of this supplement on academic achievement and their safety.

Testing the validity and reliability of the questionnaire

The questionnaire was designed by the investigators in both Arabic and English languages. A pilot study was conducted by sending the links of the questionnaire to 30 participants with a subsequent recording of their feedback about the questions and the answer options to assess its face validity and to verify the clarity and simplicity of all questions and options for answers. After receiving feedback from the pilot study participants, a few questions were slightly changed. The questionnaire was then sent to gather data.

Statistical analysis and data management

Microsoft Excel (version 2019; Microsoft Corp., Redmond, United States) was used to extract data from Google Forms, and the IBM SPSS Statistics software (version 25; IBM Corp., Armonk, United States) was utilized to transfer the data for statistical analysis. A p-value <0.05 was considered statistically significant based on the 95% confidence level.

Respondents who mentioned that they lived outside KSA and had chronic illnesses or were receiving chronic drug therapies were excluded from the study. The collected data were explained utilizing descriptive statistics and presented as frequencies (n) and percentages. The Chi-square and Fisher's exact tests were used to explore the association between dietary supplement use and academic ability. Fisher's exact test was used whenever the assumptions of the chi-square test were violated, i.e., when any of the cell values were equal to zero or more than 20% of the projected values were less than five. Logistic regression was employed to assess the association between the studied parameters and academic performance.

Ethical consideration

The objective and scope of the study were explained at the outset of the questionnaire, along with a request for voluntary participation. The participants' names, phone numbers, and other sensitive personal information were not gathered in order to protect their confidentiality. All data was kept confidential and used only for research purposes. Dr. Soliman Fakeeh Hospital's Institutional Review Board granted ethical permission prior to the start of the study (permission number: 331/IRB 2022).

## Results

Survey response rate

Out of 650 students who began the survey, 513 participants completed it, yielding a 78.9% survey response rate. Other responses were excluded from the study because they were either incomplete, lived outside KSA, had chronic medical illnesses, or were receiving chronic drug therapies. 

Demographic features

Age distribution revealed that most of them, 414 (80.7%), were between 18-25 years old. The study included 273 (53.2%) females and 240 (46.8%) males. Regarding nationality, Saudi participants were in the majority, around 409 (79.7%). Table 1 provides comprehensive socio-demographic details.

**Table 1:** Socio-demographic characteristics of participants

Socio-demographic Data		Frequency (n)	Percentage (%)
Age	18 to <25	414	81
	25 to <35	73	14
	≥35	26	5
Gender	Male	240	46.8
	Female	273	53.2
Nationality	Non-Saudi	104	20
	Saudi	409	80

Academic characteristics 

Medical students represented 343 (66.9%) of the studied population, whilst non-medical students constituted 170 (33.1%). Undergraduates were in the majority, 474 (92.4%), while postgraduate students were around 61 (11.9%). Regarding the participants’ academic achievements, they were classified into high or low performers based on their grades in the previous three years. Those with grades A and B were considered high performers, while those with C, D, or F grades were considered low performers. Results revealed that 217 (42.3%) participants were low performers, while 296 (57.7%) were high performers (Table 2).

**Table 2:** Academic characteristics of participants

Academic Data		Frequency (n)	Percent (%)
Type of your study	Medical	343	66.9
	Non-medical	170	33.1
Academic year	Year 1	65	12.7
	Year 2	50	9.7
	Year 3	97	18.9
	Year 4	137	26.7
	Year 5	64	12.5
	Year 6	39	7.6
	Postgraduate	61	11.9
Achievability	High performer	296	57.7
	Low performer	217	42.3

Dietary supplement use among participants

**Figure 1:**
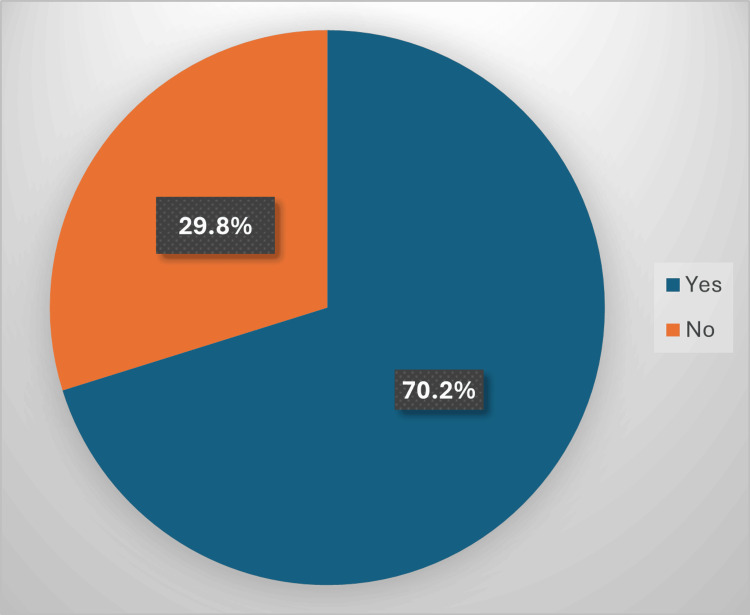
Prevalence of dietary supplement use among participants (n=513)

Most of the participants, 360 (70.2%), were using dietary supplements, while 153 (29.8%) were not taking any supplements (Figure 1).

A chi-square test of independence was used to assess the relationship between the study type, whether medical or non-medical, and the use of dietary supplements. It revealed no statistically significant association between the type of the study and the use of supplements (the p-value was 0.58, which is greater than the significant threshold of 0.05) (Table 3).

**Table 3:** Relation between supplement use and study type of participants p>0.05 indicates not significant. df: Degree of freedom

Type of Study	Supplement Use		Total	Chi-square	df	p-value
	Yes, n (%)	No, n (%)				
Medical	238 (46.4%)	105 (20.5%)	343 (66.9%)	0.307	1	0.58
Non-medical	122 (23.8%)	48 (9.4%)	170 (33.1%)			
Total	360 (70.2%)	153 (29.8%)	513 (100%)			

Among students who were taking supplements, caffeine was the most widely used supplement (106, 29.4%), followed by omega-3 fatty acids (47, 13.1%). Other supplements, such as multivitamins, creatine, curcumin, and *Ginkgo biloba*, were used at lower rates. Moreover, 141 (39.2%) of participants reported intake of supplement combinations. The frequency of supplement use revealed that most participants (266, 73.9%) were using supplements daily, while 50 (13.9%) used them weekly, and only 44 (12.2%) rarely received the supplements. Most participants (132, 36.7%) used the supplements for a long duration (more than six months); 117 (32.5%) used them for a period between one and three months, and a few participants used them for less than one month (Table 4).

**Table 4:** Type, frequency, and duration of supplement use among participants who were taking dietary supplements (n=360)

Dietary Supplement Use		Frequency (n)	Percent (%)
What is the type of supplement you are taking?	Caffeine	106	29.4
	Creatine	11	3.1
	Curcumin	6	1.7
	Ginkgo biloba	9	2.5
	Omega-3 fatty acid	47	13.1
	Multivitamins	7	1.9
	Other supplements	33	9.2
	Combination of supplements	141	39.2
Frequency of supplement intake	Daily	266	73.9
	Weekly	50	13.9
	Rarely	44.0	12.2
Duration of using the supplement	<1 month	42	11.7
	1 to <3 months	117	32.5
	3 to <6 months	69	19.2
	>6 months	132	36.7

**Figure 2:**
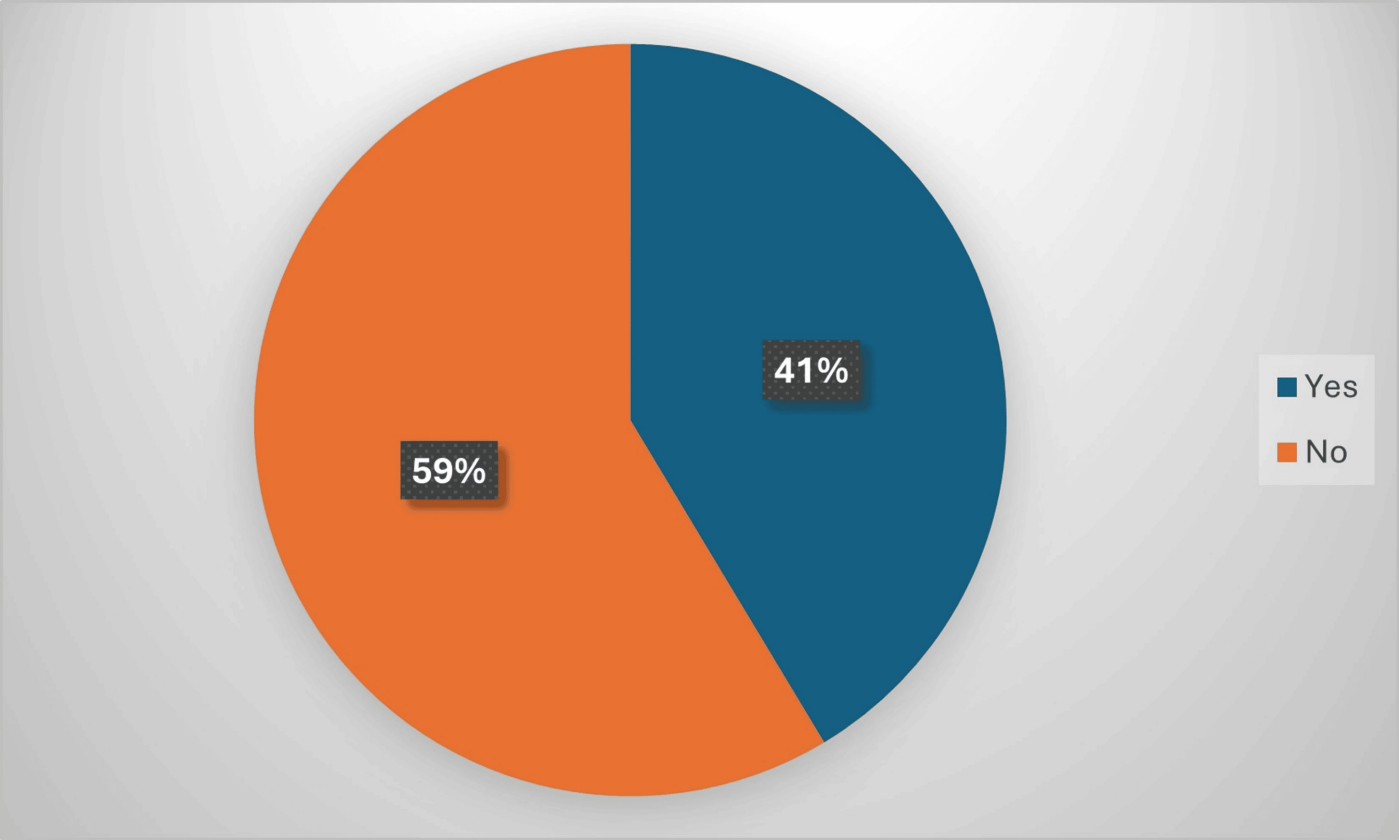
Prevalence of supplement-induced adverse events among supplement users in the studied group (n=360)

Among participants who were using dietary supplements, 149 (41.3%) reported experiencing supplement-induced adverse events, while 211 (58.6%) did not experience any adverse events (Figure 2).

Types of supplement-related adverse events 

**Figure 3:**
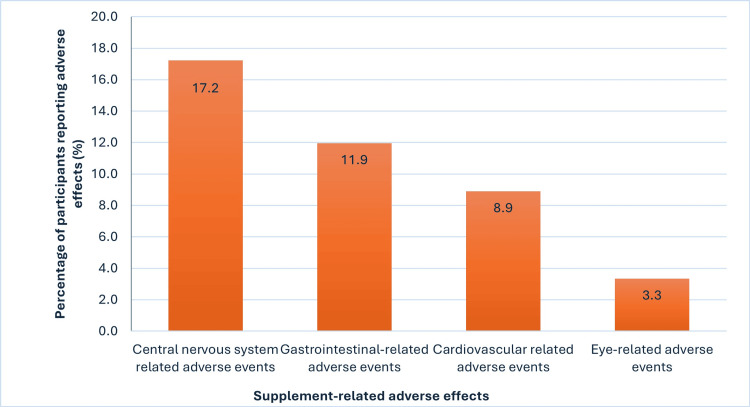
Prevalence of supplement-induced adverse events among supplement users (n=360)

The most frequently reported supplement-induced negative effects were central nervous system problems, reported by 62 (17.2%) supplement users, such as headaches, sleeplessness, irritability, or dependency, followed by gastrointestinal symptoms that occurred in 43 (11.9%) participants receiving supplements, like nausea, vomiting, diarrhea, or constipation. Around 32 (8.9%) reported problems were cardiac manifestations, namely palpitation and blood pressure changes. The least commonly reported were eye-related adverse events, with 12 (3.3%) participants experiencing blurring of vision and eye congestion (Figure 3).

Knowledge about the benefits and risks of dietary supplements

**Figure 4:**
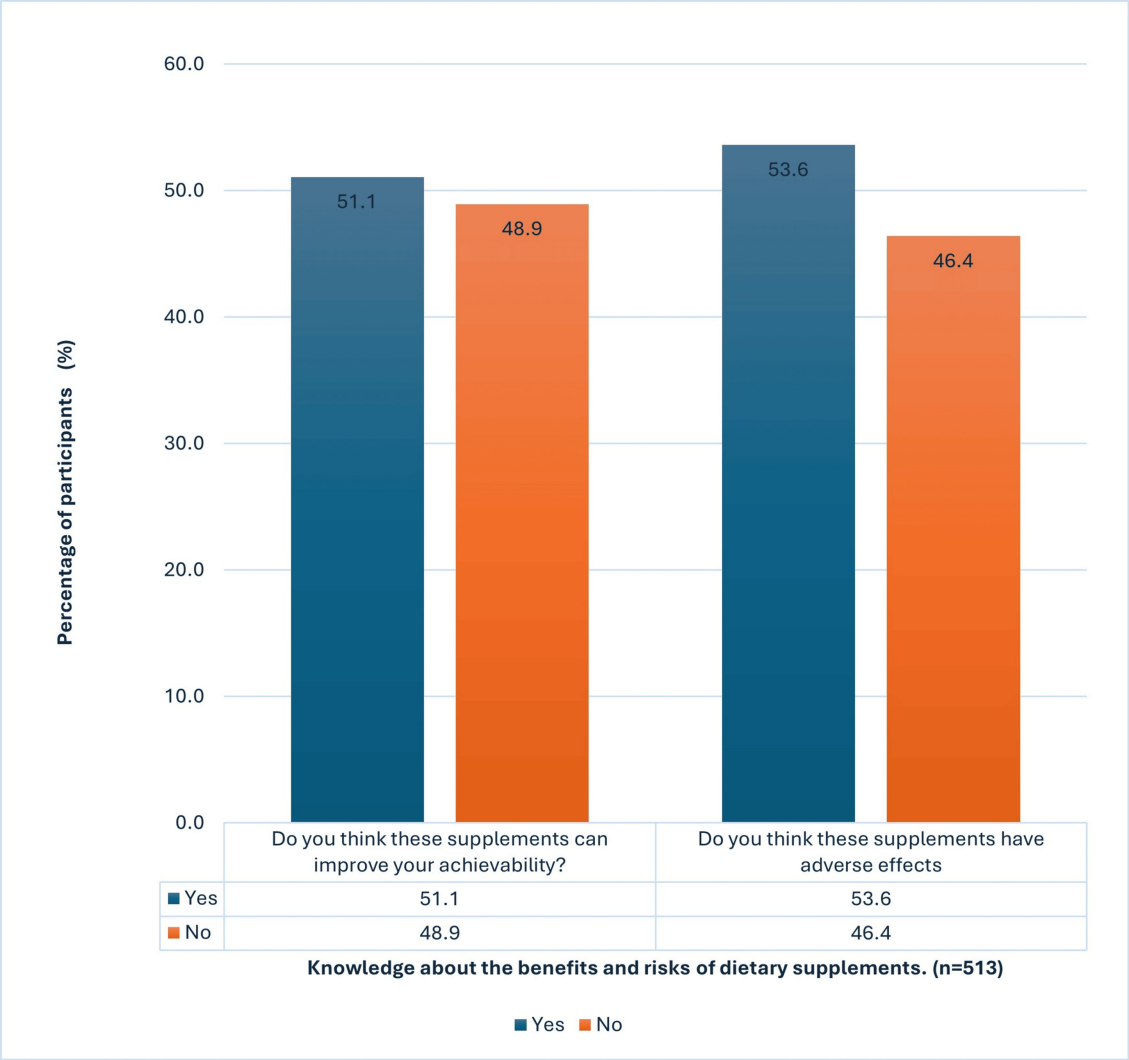
Knowledge about the benefits and risks of dietary supplements (n=513)

Of the enrolled cohort, 262 (51.5%) believed supplements could improve their achievability, while the remaining 251 (48.9%) didn’t hold this belief. Conversely, 275 (53.6%) believed supplements could have adverse events, while 238 (46.4%) considered dietary supplements not to carry any risk of adverse events, as illustrated in Figure 4.

Association between various supplement types and academic achievement

The Chi-square test was used to examine the association between dietary supplement use and academic ability. Fisher’s test was employed when the Chi-square test was violated. The results showed no statistically significant relationship between dietary supplement use and the academic performance of the students in the studied population (p>0.05) (Table 5).

**Table 5:** Association between various supplement types and academic achievement p>0.05 indicates not significant. df: Degree of freedom

Supplement	High Performers, N (%)	Low Performers, N (%)	Total	Chi-square	df	p-value
Caffeine	66 (62.3%)	40 (37.7%)	106 (100%)	5.482	8	0.705
Fish oil - omega-3 fatty acids	25 (53.2%)	22 (46.8%)	47 (100%)			
Other supplements	21 (63.6%)	12 (36.4%)	33 (100%)			
Combination of supplements	87 (60.0%)	58 (40.0%)	145 (100%)			
No supplements	77 (51.7%)	72 (48.3%)	149 (100%)			
Supplement	High Performers, N (%)	Low Performers, N (%)	Total	Fisher's Test Value	df	p-value
Creatine	8 (72.7%)	3 (27.3%)	11 (100%)	5.605	-	0.7
Curcumin	3 (50%)	3 (50%)	6 (100%)			
Ginkgo biloba	5 (55.4%)	4 (44.4%)	9 (100%)			
Multivitamins	4 (57.1%)	3 (42.9%)	7 (100%)			
Total	296 (57.7%)	217 (42.3%)	513 (100%)	-	-	-

Association between the studied factors and academic performance 

Logistic regression analysis showed no significant correlation between academic achievement and the majority of the parameters examined, except for gender, where female individuals had a considerably higher chance of achieving higher academic performance (p=0.019) (Table 6).

**Table 6:** Association between each of the studied factors and academic performance * indicates statistically significant correlation. B: Beta coefficient, which represents the strength and direction of the association; S.E.: Standard error, which indicates the variability or precision of the beta coefficient; Wald: Wald Chi-square test, which is a test statistic used to assess whether the beta coefficient is significantly different from 0 (i.e., whether the predictor has a significant impact on the outcome); df: Degree of freedom; Sig.: Significance or p-value (a p-value <0.05 indicates a significant association); Exp(B): Exponentiated beta or odds ratio

Studied Factors	B	S.E.	Wald	df	Sig.	Exp(B)
Age	0.322	0.190	2.890	1	0.089	1.380
Gender	-0.451	0.192	5.500	1	0.019*	0.637
Nationality	0.135	0.233	0.334	1	0.563	1.144
Type of study	0.020	0.203	0.009	1	0.923	1.020
Academic year	-0.020	0.058	0.125	1	0.723	0.980
Intake of dietary supplements	0.110	0.234	0.221	1	0.638	1.116
Supplement type	0.028	0.035	0.666	1	0.415	1.029
Duration of receiving supplements	0.017	0.078	0.047	1	0.829	1.017
Frequency of receiving supplements	-0.026	0.126	0.043	1	0.837	0.974
Considering supplements improve the achievement	-0.056	0.191	0.088	1	0.767	0.945
Considering supplements carry a risk of adverse events	-0.067	0.200	0.110	1	0.740	0.936
Experience supplement-induced adverse events	0.077	0.222	0.120	1	0.729	1.080
Constant	-0.924	0.993	0.867	1	0.352	0.397

## Discussion

The purpose of the current study was to assess the dietary supplement utilization among students in KSA and how these supplements affected their academic performance. The findings revealed a large prevalence of dietary supplement intake among the studied group, with about 360 (70.2%) participants taking supplements. Most supplement users were taking a combination of supplements, while caffeine was the most used single supplement. Also, the majority of supplement users were taking them daily. Although coffee, which is known to have stimulant action, was commonly consumed by the participants, there was no discernible link between supplement usage and academic achievement. Remarkably, there was no significant association between academic achievement and the usage of any of the mentioned nutritional supplements among participants in our study. No statistically significant difference was identified between the prevalence of using supplements between medical and non-medical students as well.

These results align with those of previous studies by Mahoney et al. (2019) [19], where it was found that more than 90% of students in the United States consume caffeine to enhance their performance, mood, wakefulness, concentration, and physical endurance. Furthermore, a recent study carried out in KSA found that 20% of students take more caffeine than is advised daily [20]. Comparable findings were reported by Vidović et al. (2022) [21] who identified a high prevalence of supplement use among university students in Belgrade, reaching 55.7% of students. Nevertheless, they observed a higher frequency among medical students, which runs counter to our findings.

Adverse events associated with supplements were reported by 147 (41%) supplement users in the current study. Central nervous system side effects, including headaches, irritability, sleeplessness, and dependency, were the most common, followed by gastrointestinal and cardiovascular issues. These results are consistent with a prior study carried out by Eltyeb et al. (2022) in Jazan [22] who evaluated the effects of caffeine consumption and stated that headache, nausea, and tachycardia are the most encountered adverse events.

The unwanted effects reported by the studied population could be attributed to the intake of high doses of caffeine or inappropriate use of other supplements. This comes in line with a recent study conducted in KSA [23] that revealed a high prevalence of self-medication among adults and a high frequency of unsafe medication-related behaviors, such as taking them after their expiration date, without verifying the expiration date, or without reading the directions. Furthermore, a study carried out in the United States found that adverse events associated with supplements are frequent, especially in the central nervous system, gastrointestinal tract, and cardiovascular system.

The findings of our study revealed that the participants' achievability was unaffected by their supplement usage, despite the high prevalence of supplement intake and the substantial risk of supplement-related adverse events. All studied parameters didn’t affect academic achievability, except for gender, where the female sex was associated with higher academic performance. These results are consistent with a recent systematic analysis that examined 50 research studies to determine the impact of iron supplementation on intelligence quotient (IQ), memory, and recall and discovered no relationship between them [7]. An additional intervention trial conducted in India found that while ragi porridge, an iron-rich Indian recipe, might raise hemoglobin levels; however, it did not affect the participants’ academic performance [24].

Conversely, a six-month interventional trial in India found that participants' attention, memory, and reaction time were all enhanced by an iron-fortified pearl millet [25]. This discrepancy can be explained by the participants' varying ages - the study included adolescents between the ages of 12 and 16 years - as well as the differences in the living standards between populations in KSA and India, where malnutrition and poor socio-economic status are more common in India. That is why in the Indian population, iron administration corrected the anemia and improved the cognitive skills.

An evaluation of the participants' knowledge of the advantages and risks of dietary supplements showed that roughly 262 (51.1%) thought that taking supplements could help them do better academically; however, this was not shown in this study. About 238 (46.4%) considered supplements completely safe with no risk of side effects, although the supplement-associated adverse events were highly prevalent among the studied group and in other studies [26,27]. This demonstrates the lack of correct knowledge regarding dietary supplements.

The lack of beneficial effect of supplements on participants' academic achievements and poor knowledge about their benefits and risks, despite the high cost of dietary supplements worldwide - the global market for brain health supplements was estimated to be worth USD 7.6 billion in 2021, with anticipation to increase to USD 15.59 billion by 2030 [28] - makes it clear that more awareness is needed to enhance rational use of the dietary and brain supplements. Campaigns to raise public and students' awareness of the advantages and dangers of dietary supplements are highly recommended.

Limitations

This study used an online self-taken questionnaire as a data collection tool. This resulted in some limitations. For example, participants with proficiency in Arabic or English reading only could have been recruited; however, those with language barriers, like those who were proficient only in other languages, such as Urdu, Hindi, or Tagalog, which are spoken by some resident populations in Saudi Arabia, couldn’t participate in this study. Although there was a minimal chance that this limitation would have affected the study, it might have left out some population members.

The questionnaire was distributed via social media platforms, so it was out of reach for individuals with no access to the internet or social media, particularly in more rural areas. Although there is very little risk associated with this restriction because internet access is widely available in KSA, particularly for students, some people may have been left out, especially those from low-income families.

The risk of misinterpretation of some questions was present because the questionnaire was self-administered. However, considering the questions' straightforward language and the validity assessment of the questionnaire prior to the start of the study, the degree of misunderstanding is probably quite low.

Additionally, the convenient sampling technique used in the current research means that the sample does not completely represent all of the students in KSA. Given that the sample might have been skewed toward students who were more tech-savvy, this limitation is noteworthy.

That is why the findings of this study cannot be generalized to the whole population, except after more research is conducted to confirm the findings.

Furthermore, the cross-sectional study design makes it difficult to pinpoint exactly how the many variables under investigation relate to one another. Although this is a typical drawback of cross-sectional research, the results offer insightful preliminary information. To validate these findings, more studies using experimental or longitudinal designs will be required.

Additionally, the idea of implementing programs to raise student understanding of the advantages and risks of dietary supplement use can improve students' health and lower the negative impacts and expenses associated with supplement use.

## Conclusions

The study showed that students in KSA commonly used dietary supplements, with caffeine being the most consumed. No discernible influence on academic performance was found, despite the high prevalence of supplement use and the related negative adverse events, which include central nervous system, gastrointestinal, and cardiovascular disturbances. Participants' knowledge about the benefits and safety was inappropriate, as they overestimated the supplement's impact on their academic achievability and didn’t realize the risks of adverse events. Notably, students' inadequate awareness about dietary supplements highlights the need for awareness programs to encourage careful and informed use. It is crucial to note that academic performance was not compared before and after supplement use in this study. To fully comprehend the true effect of supplements on academic performance, further research using a longitudinal design is required. 

## Appendices

**Table 7:** Questionnaire for assessing the association between dietary supplement use and academic achievement

Section	Question	Options
Demographics	What is your age?	18 to <25
		25 to <35
		>35
	What is your sex?	Male
		Female
	What is your nationality?	Non-Saudi
		Saudi
	Where do you currently reside?	Inside the Kingdom of Saudi Arabia
		Outside the Kingdom of Saudi Arabia
Medical and medication history	Do you have any chronic illness or take any medications regularly?	Yes, I have a chronic illness
		Yes, I take medications regularly
		No, I do not have a chronic illness or take medications regularly
Academic information	What is your academic program or field of study?	Medical studies
		Non-medical studies
	Which academic year are you currently in?	Year 1
		Year 2
		Year 3
		Year 4
		Year 5
		Year 6
		Postgraduate student
	How would you rate your academic performance over the past three years?	High performer (Grade A or B)
		Low performer (Grade C, D, or F)
Dietary supplements use and knowledge	Are you currently taking any dietary supplements?	Yes
		No
	Which supplements are you currently taking? (Select all that apply)	Caffeine
		Creatine
		Curcumin
		Ginkgo biloba
		Omega-3 fatty acids
		Multivitamins
		Other (please specify)
	How long have you been taking these supplements?	Less than one month
		1 to <3 months
		3< to 6 months
		More than 6 months
	How often do you take dietary supplements?	Daily
		Weekly
		Rarely
	Have you experienced any adverse effects from these supplements?	Yes (please specify)
		No
	Do you think these supplements can improve your achievability?	Yes, supplements can improve academic achievability
		No, supplements can’t improve academic achievability
	Do you think these supplements have any adverse events?	Yes, supplements can cause adverse events
		No, supplements are completely safe.
